# Ex vivo liquid core fiber photometry with high-resolution 3D printing

**DOI:** 10.1016/j.snr.2024.100227

**Published:** 2024-07-20

**Authors:** Yu Chang, Can Wang, Ke Du

**Affiliations:** a Department of Chemical and Environmental Engineering, University of California, Riverside, CA 92521, USA; b Department of Mechanical Engineering, Rochester Institute of Technology, Rochester, NY 14623, USA

**Keywords:** Optofluidic, Stereolithography, Ex vivo, Photometry

## Abstract

High resolution 3D printing emerges as an alternative to microfabrication due to its fine resolution along with one-step manufacturing. Thus, it is broadly used in many fields, such as biological and chemical applications. We introduce such a technique to the design of the optofluidic probe by integrating optics and microfluidics as an ex vivo liquid core fiber photometry. We build the optofluidic probes with various T-shapes and conduct the transmission measurements and the ray tracing simulations, where the results are comparable. Through the transmission and fluorescence measurements, we obtain optimized curl T-shape dimensions of 524 μm wide, ~50 μm thick, and 350 μm long with longitudinal spaces between them of 260 um. Furthermore, a heightened level of complexity in structure, characterized by a feature size of 25 μm, is attained through the improvement process. We conclude the feasibility of this optofluidic system with two applications: the in vivo-like setting consisting of thyroid biopsy training phantom and human plasma and the ex vivo-like setting consisting of the mice brain slices stained with wheat germ agglutinin (WGA). This prototype is an important step of establishing a 3D printing optofluidic applications for various in vivo research.

## 1. Introduction

Optofluidic platforms are interdisciplinary between the conventional light-guiding device and the microfluidic system [1]. Due to the combination of both, the platforms are re-configurable in terms of the various featured optical elements and microchannels, thus providing a variety of solutions to study light-matter interactions between the single or multi-species of molecular targets and the applied electromagnetic wave [2,3]. The technology has enabled applications in microreactors [4], optical tweezers [5], and biosensing fields [6], Furthermore, the optical elements are not limited to traditional photonics but can be the aqueous medium, which is used as a molecular cargo or the desired sensing environment. The characteristics of microfluidics, such as miniaturization and versatility, could further potentiate the sensing performance and flexibility. The optofluidic probe, one of the optofluidic systems, has demonstrated its value in vivo and ex vivo applications such as the neural probe [7,8], coherent tomography imaging [9], intracellular delivery [10], temperature sensor [11], DNA detection [12], and even integrated multifunctional device [13].

Micro-electromechanical systems (MEMS) paved a broad way for manufacturing microfluidic devices [14,15]. With the benefits of miniaturized volume and compact arrangement, the sample volume could be immensely reduced, and the light guiding or mechanical operation section can be integrated on a small chip. However, the process usually calls for a series of manufacturing steps in a clean room environment, which are time-consuming and less cost-efficient. The morphological limitations associated with this approach, which is typically built in a planar pattern, may present challenges in enhancing design capabilities. In addition, to combine the optical elements and the microfluidic chips, additional sealing and binding are necessary. Alternatively, additive manufacturing, also known as 3D printing technique, is a novel candidate for constructing intricate objects in one or a few steps. The technology facilitates the creation of arbitrary geometrical designs with feature sizes that are comparable. Nowadays, microstereolithography (μ-SLA) [16], microscale selective laser sintering (μ-SLS) [17], and two-photon polymerization (2PP) [18], the three photoactivated-based 3D printing methods, are capable of achieving the feature sizes in the proximity of 1 μm [19], 5 μm [20], and less than 100 nm [21], respectively. High resolution 3D printing has enabled several applications such as lens-in-lens probe for in vivo endoscopy [22] and biomimetic surface for liquid manipulation [23].

In our previous work, a fully enclosed optofluidic channel with hydrophobic microstructures was enabled by high resolution 3D printing [24]. The technique is more affordable and has less power consumption while keeping an efficiently small feature, enabling an aspect ratio of more than 40:1. Here, we design various “T-shape” based micro-structured chips as optofluidic probes prototypes. In this work, we improved our optofluidic chip by creating more clearances in both axial and radial directions, along with a reduced feature size of 25 μm. Additionally, we have introduced secondary structures featuring a more intricate morphology that is challenging to manufacture through conventional cleanroom processes. The design guideline is initiated from the ray tracing simulation analysis and verified by the transmission and fluorescence measurements. To demonstrate the potential applications, we implemented the in vivo-like fluorescence measurements in a mimic environment, represented by a training phantom and human plasma. The results agree with the in vitro analysis. In addition, the successful identification of fluorescence signals on the stained C57BL/6 mice brain slice proves that the probe can be used to observe a fixed fluorescence source. Our results show that the proposed probe can be potentially used to fulfill both in vivo and ex vivo fluorescence measurements as a photometry.

## 2. Experimental section

### 2.1. Optofluidic chip fabrication

High resolution 3D printing set and photocurable resin (HTL) were chosen to fabricate the optofluidic chips. The printing set contains a printer (MicroArch^®^ P140, Boston Micro Fabrication), a wash station (Form wash, Formlabs), and a UV post-curing station (Form cure, Formlabs). The printer is capable of printing the designed microstructures with an optical resolution of 10 μm. After printing, the samples were soaked in isopropyl alcohol and washed in the wash station for 10 min. The washed pieces were baked at 45 °C under UV exposure in the post-curing station for 7 min. All of the microstructures were coated with PTFE (Teflon AF 6 %, Chemours) via dip coating, followed by a baking step at 110 °C for 3 h.

### 2.2. Transmission measurements

The overall setup is composed of an LED driver connected to a fiber-coupled LED of 405 nm (M405FP1, Thorlabs), a lab-printed transmission platform, and a spectrometer (Ocean Insights). For transmission measurement, an optical fiber connecting a fiber-coupled LED was inserted into the fiber coupling hole of the micro-structured chip. A tubing was also connected to the chip for liquid injection. A transmission tank was printed by 3D printing (Saturn 2, Elegoo) with a dimension of 38 mm ×38 mm × 13 mm, with an open hole in the bottom for signal collection. The tank was then filled with DI water through the chip, where the chip was placed and confined in the center of the tank. Another fiber was centered and mounted in the bottom of the tank for detection. A schematic of the setup is shown in Fig S1.

### 2.3. Transmission simulation

A commercial software (TracePro^®^, Lambda research) based on the Monte Carlo method was applied for the analysis. Three objects were arranged in the simulation, including a light source, an absorber (receiver), and the microstructured chip. The light source was assumed to be Cree Xlamp, with 1500 total rays. The absorber was assumed to be perfect, which indicates no reflectance or transmittance. The optical properties of HTL are based on the reported studies [25]. The medium within the chip was assumed to be DI water. The illuminance maps for absorbed flux on the surface of the absorber under the same incident rays were collected for further analysis.

### 2.4. Fluorescence measurement

For fluorescence measurement, the detection fiber was placed in the fiber coupling hole instead of in the bottom of the tank. The tank was filled with Qdots (605 ITK^™^, Thermo Fisher Scientific) with a concentration ranging from 2.5 to 20 nM. For the thyroid biopsy training phantom sensing (EDM Medical Solutions), Qdots were filled into the phantom with a volume similar to a small mouse brain (~508 mm^3^) [26]. The DI water was replaced by human serum (H4522, MilliporeSigma). Both fluorescence measurement setups were designed to approximate an in vivo environment.

### 2.5. Brain slice staining

A non-implanted protocol was presented to acquire authentic data based on brain samples and in anticipation of the future animal testing phase. The frozen brain slices of C57BL/6 mice (coronal cross-orientation, Zyagen) were first fixed with 4 % Paraformaldehyde (Thermo Fisher Scientific) for 15 min to preserve the tissue structure. Subsequently, the sections were incubated with the wheat germ agglutinin (WGA) staining solution in a dark environment at room temperature for 30 min. To prepare the staining solution, lyophilized WGA lectin powder labeled with Alexa Fluor^®^ 350 and 488 (Thermo Fisher Scientific) was diluted with 1× PBS to achieve a concentration of 100 μg ml-1 for each dye. Following the staining process, the images of the brain slices were captured under a fluorescence microscope, and the stained pieces were ready for use.

## 3. Results

**Fig. 1.**
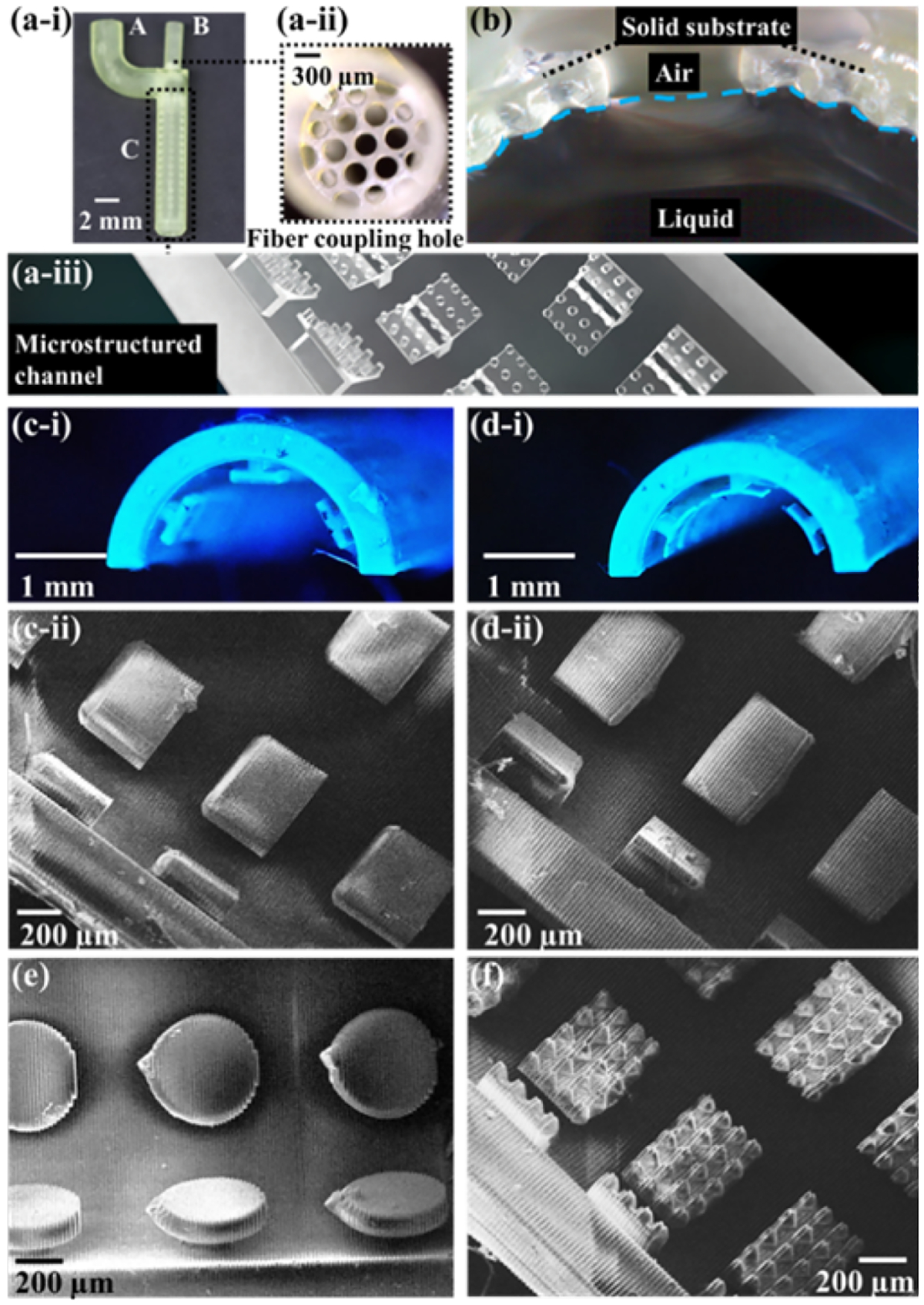
(a-i) Symbol A, B, and C represent the inlet for liquid, fiber coupling hole, and microstructured channel, respectively. (a-ii) A porous layer contains several holes with diameters of 300–400 μm in the bottom of the fiber coupling hole. (a-iii) The optofluidic channel with hierarchical microstructures on the inner sidewall. (b) The interface between the liquid core and the cladding layer with hierarchical microstructures. Micrograph of the optofluidic chip with (c-i) Flat T (d-i) Thin-curl T microstructures. SEM image of the optofluidic chips with (c-ii) Flat T (d-ii) Thin-curl T (e) Circular T and (f) Curl T-protrusions microstructures.

In the initial stages of optofluidic probe development, an evaluation was conducted on micro-structured optofluidic chips with regard to several aspects, including printing quality, ray tracing simulations, transmission measurements, and fluorescence measurements. The printed optofluidic chips consist of three main components, as illustrated in Fig 1a–i: an inlet for the injection of liquid, a hole designed for fiber coupling, and a microstructured channel. Notably, there is a porous layer present at the bottom of the fiber coupling hole, as depicted in Fig 1a–ii. The dimensions of the porous film hole, the diameter of the fiber coupling hole, and the length of the microstructured channel were investigated during the design phase. In our previous investigation, a T-shaped structure was devised, showing excellent light-guiding capabilities and mechanical robustness compared to the other designs, such as micropillars and micro-gratings [24]. The hydrophobic micro-structures, presented in Fig 1a–iii, give rise to a three-phase interface involving liquid, solid printing material, and air, as shown in Fig 1b [27].

The T-shape morphology and its associated dimensions, encompassing width, thickness, length, and inter-spacing, underwent variation and thorough investigation. Four distinct morphological configurations of these microstructures are depicted in Fig 1c–i through Fig 1f, respectively. Fig 1c–i and Fig 1c–ii show T-shape structures featuring flat heads with a thickness of 90 μm, while Fig 1d–i and Fig 1d–ii present structures with thin and curved heads having a reduced thickness of 25 μm. The disparity in the curvature of the T-shape heads is evident when comparing Fig 1c–i to Fig 1d–i. Additionally, Fig 1e and Fig 1f display morphologies characterized by circular T-shape structures and curved T-shape (Curl T) structures with protrusions on the top, respectively. For the curl T structures with protrusions, the thickness of the T-shape head tapers gradually from the center to the periphery, resulting in a transition from 75 to 25 μm in thickness. Both the diameter and the height of each protrusion measure 50 μm. Notably, while certain distortions were observed in the printed microstructures, such imperfections remain within an acceptable range. This is attributed to the substantial differences in dimensions among the various design configurations, ultimately exerting a more dominant influence on the performance of light guidance.

**Fig. 2.**
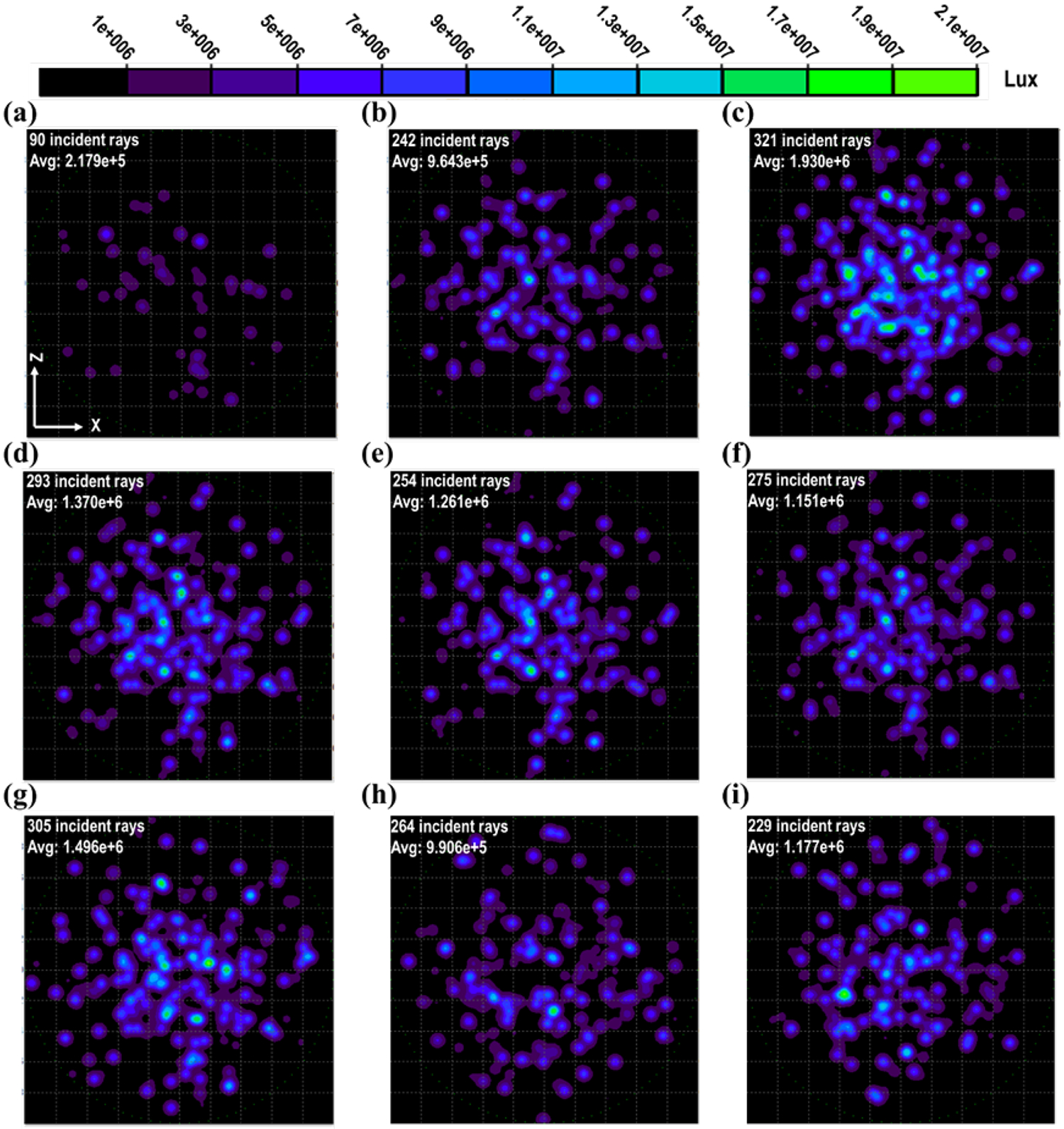
The ray tracing simulations for the different dimensions of T-shape microstructures are demonstrated as illuminance maps for absorbed flux in global coordinates. The dimensions are collected in Table 1. (a) Flat channel (without microstructures); (b) Wide T; (c) Narrow T; (d) T with a width of 393 μm; (e) Thick T; (f) T with a width of 524 μm; (g) Short length T; (h) Flat T; (i) Circular T.

Ray-tracing simulations were employed to optimize the transmission performance of the designed optofluidic chips. In these simulations, the length of the optofluidic chip remained constant, while the structures’ dimensions and inter-spacing (Fig S2) were varied, as detailed in Table 1. The simulation setup involved the creation of a light source positioned within the fiber coupling hole of the chip, with an absorber situated beneath the chip. Multiple illuminance maps displayed in Fig 2 were generated to represent the absorbed flux based on this simulation arrangement. Our initial simulation pertained to the transmission within a flat channel devoid of microstructures (Fig 2a). This configuration exhibited the least incident rays or flux upon the absorber’s surface. In comparison, a superior transmission result was achieved when employing the curl T-structures with a width of 654 μm, as shown in Fig 2b, which was the widest among the designs. Further enhancing the transmission performance was possible by using a curl T head with a width of 131 μm while maintaining other dimensions constant (Fig 2c). Fig 2d, on the other hand, displayed an intermediate number of incident rays and the intensity due to a moderate curl T width of 393 μm.

Nevertheless, marginal variations in both the number of rays and intensity were observed in the microstructures depicted in Fig 2e and Fig 2f, as compared to the more pronounced differences observed between Fig 2c and Fig 2d in relation to the widest case. These microstructures were characterized by increased thickness and width, resulting in greater absorption due to the higher solid fraction within the complex cladding. Based on our experience, a thinner T head tended to reflect more light beams towards the core, attributable to the “air mirror” effect [28,29]. In the comparison of Fig 2e with Fig 2f, it is discerned that the correlations between the quantity of rays and the intensity may not invariably be positive. This discrepancy arises from the fact that certain rays might undergo a higher frequency of absorption and scattering events throughout their propagation. In Fig 2g, a reduction in the length of the microstructures from 350 μm to 150 μm was implemented, leading to a 40 % decrease in solid contact area compared to the dimensions in Fig 2d, leading to a higher transmission result. Fig 2h and Fig 2i introduced dissimilar morphologies resembling those shown in Fig 1c–ii and Fig 1e, respectively, to elucidate the influence of cross-section shape. Theoretically, a round core is inclined to confine light beams along the center axis due to its symmetry. This was confirmed by comparing the transmissions of Fig 2h and Fig 2f; despite the latter having a wider T, representing a 22 % larger solid contact area, it still demonstrates better performance. Fig 2i had the minimum number of incident rays among the microstructured channels. The structure demonstrated a solid contact area that as 14 % smaller and an overall solid fraction that experienced an almost 34 % reduction when juxtaposed with the curl-T configuration used in Fig 2b. In contrast, the structure adopted in Fig 2f showed only a modest 20 % reduction in solid fraction compared to that featured in Fig 2b. However, the intensities of Fig 2f and Fig 2i are rather close. This indicates the contribution of near-round cross-section results in Fig 2f Consequently, the subsequent experiments would be based on the curl T morphology.

The analysis of simulations indicates that for efficient support of the liquid and minimization of absorption loss, the curl T heads should be thin, possess a short longitudinal dimension, and have a reasonably wide circumferential dimension. Subsequently, we proceeded to print the first seven curl T designs labeled T-1 to T-7, with their respective dimensions outlined in Table 2.

**Fig. 3.**
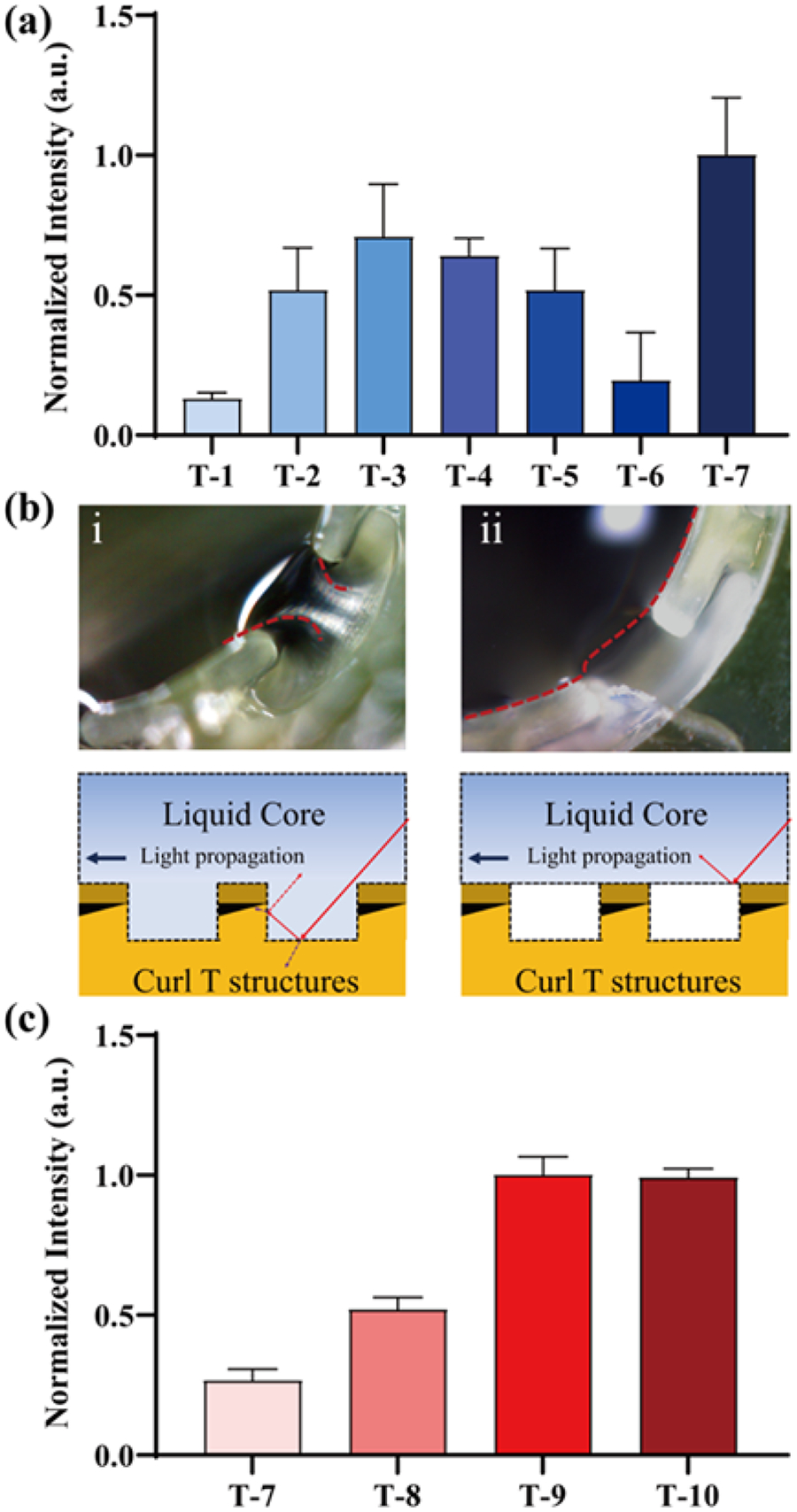
The transmission measurement of the seven designs with a space of 350 μm. The intensities were normalized by dividing each intensity by T-7. (b-i) Micrograph showing the liquid falls off in the longitudinal gap, causing significant signal loss. (b-ii) The liquid is well confined. (c) The transmission measurement of the four designs with varied spaces. The intensities were normalized by dividing each intensity by T-8.

A transmission measurement was conducted on these chips to determine the most suitable design configuration using an arrangement similar to the one employed in the simulation. In Fig 3a, the transmission intensity of T-1, which is a flat chip, is observed to be the lowest, aligning with our simulation results. T-2 and T-3 share identical dimensions except for the width of the T head. T-3 exhibits higher transmission despite its wider head width of 654 μm. This discrepancy arises from the fact that in the case of T-2, the liquid fills the interstices between the microstructures. This led to the realization that a width of 524 μm is ideal for maintaining a circular cross-section shape, and this measurement was applied in subsequent designs (Fig S3). With a fixed width, we printed the curl T structures, each with unique characteristics: a shorter length (T-4), an extended length (T-5), and a thin-head T (T-6). Most of the microstructured chips displayed considerably higher transmission intensities compared to T-1 (the flat chip), except for T-6, as the printing geometry approached the resolution limit of the 3-D printer. It became evident that the head width of the incompletely printed T-6 was notably narrower and insufficient to support the liquid core (Fig S4). T-4 showed slightly higher transmission than T-5 due to its reduced head length, which resulted in a 50 % reduction in solid contact area. Theoretically, T-4 should have delivered superior performance based on its shorter length and slightly narrower width. However, during experimentation, there was no substantial difference between T-4 and T-3, with T-7 emerging as the most efficient design within this group. Subsequent investigation revealed that in the case of T-4 and T-7, the liquid tended to settle into the gaps in the longitudinal direction, causing a non-straight propagation boundary along the axis, as illustrated in Fig 3b–i. This, in turn, led to more diverse scattering of light beams when compared to Fig 3b–ii. Furthermore, given T-7′s moderate structural length, it was capable of mitigating the negative effects caused by the irregular longitudinal cross-section, in contrast to T-4, while still maintaining a reasonable length to prevent an excessive increase in the solid contact area, as observed in T-5. Consequently, we proceeded to investigate the spacing between the T-shape structures to optimize transmission. In Fig 3c, T-8, T-9, and T-10 were fabricated based on the design of T-7, with modifications made to the spacing and the thickness of the T-shape head. T-9 and T-10 displayed stronger transmission intensities than T-8 when a suitable spacing of 260 μm was employed. T-10 was also printed with a reduced head thickness of 25 μm, with the expectation of enhancing the number of reflected rays to the core; however, this adjustment did not yield a significant improvement. This suggests that, in this particular case, reducing the head thickness by 75 μm does not result in a significant improvement compared to the previous dimensional modifications.

**Fig. 4.**
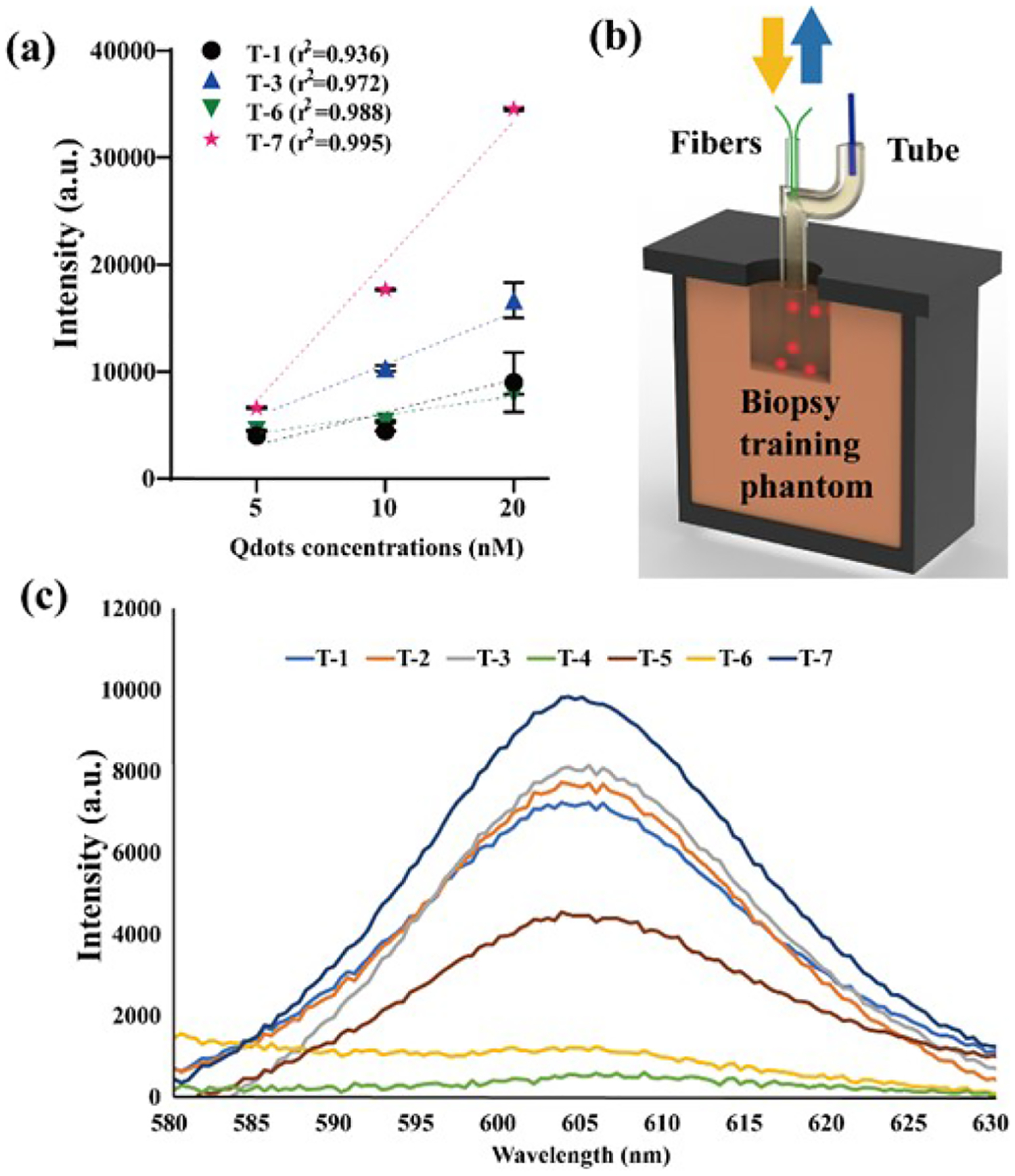
(a) The fluorescence measurement of Qdots with various concentrations. (b) The schematic represents the setup of fluorescence measurement consisting of a lab-printed tank and the thyroid biopsy training phantom. (c) The fluorescence spectrum of the 20 nM Qdots within human plasma measured with the first seven designs.

Fluorescence measurements were also conducted on the initial seven chips. In the experimental setup, the fluorescence measurement procedure paralleled that of the transmission measurement. We employed a lab-designed transmission setup, modifying it by relocating the detection fiber from the bottom to the fiber-coupling hole of the chip. The measurements encompassed Qdots with various concentrations. Qdots were selected for their exceptional brightness and wide use for live cell and in vivo imaging. Among the various designs, T-7 emerged as the best in terms of the correlation coefficient (Fig 4a). T-1, T-3, and T-6 exhibited adequate correlation coefficients, with T-3 ranking as the second-best. In general, T-7 outperformed the other designs primarily due to its judiciously printed circumferential and longitudinal cross-section shapes. Meanwhile, T-3 possessed a circular cross-section in the circumferential direction, as elucidated in the transmission measurements. The outcomes for T-1 and T-6, representing the flat channel and incomplete structures, respectively, revealed stable albeit smaller positive correlations.

To simulate a measurement environment resembling in vivo probing, we established a thyroid biopsy training phantom chamber, as depicted in Fig 3e, and substituted the DI water with human plasma. The chips exhibited comparatively lower intensities and displayed less positive correlations compared to the results obtained in the lab-printed tank setting. This deviation can be attributed to the increased variability introduced during the handling of chips that were free to move and be positioned without additional support, thus mimicking real-world operational scenarios. However, it’s worth noting that such influences tend to be insignificant when dealing with high levels of fluorescent intensities. Based on the peak intensities detected at a concentration of 20 nM, as illustrated in Fig 4c, T-7 demonstrated the most optimal performance, followed by T-3, which aligns with the outcomes from our transmission results. Additionally, T-2 secured the third position, followed by T-1, T-5, and T-6. In practice, the transmission and fluorescence measurements were conducted on the first seven chips using the lab-printed tank and the training phantom. Given the resemblance in trends observed in these experiments, it is reasonable to infer that the chips should exhibit superior light-guiding capabilities if they already showed a sufficient good performance in one of the tests. Consequently, T-9 to T-10 was developed to enhance performance based on these findings, and the transmission measurement was chosen as the verification.

**Fig. 5.**
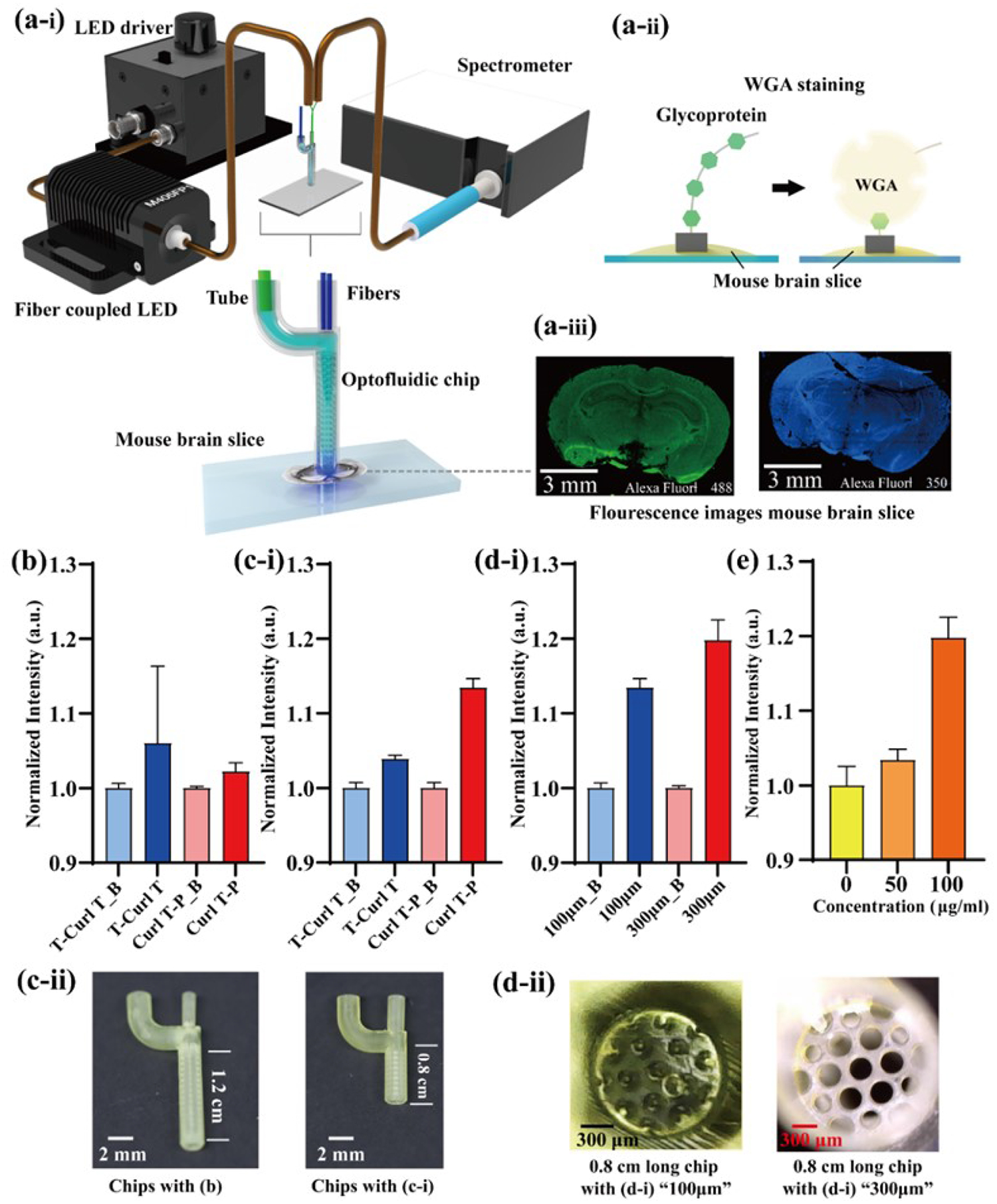
(a-i) Optical setup for the fluorescence measurements of the stained slides of C57BL/6 mice brain with optofluidics. (a-ii) The schematic of the WGA binding process. (a-iii) The fluorescence photos of the stained mice brain slices. The fluorescence measurements (Alexa Fluor^™^ 350) with the thin-curl T (T-Curl T) and curl T-protrusions (Curl T-P) are presented where the chips were built in (b) The original channel length, (c-i) The short channel length, and (d-i) The modified pores in coupling film. The sample name ends in B denotes the background intensity. (c-ii) The photos of the modified channel length (1.2 cm to 0.8 cm). (d-ii) The photos of the increased pores (100 μm to 300 μm). (e) Various dye concentrations measured by optimized chip. The intensities of (b) to (e) were normalized by dividing each intensity by its background intensity.

In the final phase, as depicted in Fig 5a–i, we demonstrated the utility of our optofluidic chip for detecting the fluorescence signal in stained C57BL/6 mice brain slices. The process of WGA staining is elucidated in Fig 5a–ii. Following the staining procedure, images of the brain slices were captured using a fluorescence microscope. In our previous assessments, we employed 50 μm holes in the porous film and a 1.2 cm long channel to avoid saturation in transmission and fluorescence measurements. For brain sensing applications, we selected 300–400 μm holes and an 8 mm long channel as the prototype. The hole size was chosen based on observations of water leakage while expanding the hole, designed to prevent fluid leakage and reduce absorbance caused by the printing material simultaneously. The length of the channel is adjusted according to the size of the mouse brain. Initially, the micro-structured probe was placed on top of the mouse brain slice rather than being immersed in a chamber, indicating that the fluorescence signal would emanate from the bottom of the probe rather than the surrounding solutions. Additionally, the fluorescence dyes used were less bright than Qdots. In both transmission and fluorescence measurements, we concluded that T-7 is the most suitable design for brain slice sensing. The performance can be further improved by introducing reasonable longitudinal and circumferential spacing (as exemplified by T-9 and T-10 in Fig 3c). In addition, we developed an alternative chip, denoted as Curl T-P structures, based on T-10, which includes thin-curl T (T-Curl T) microstructures. Curl T-P features a reduced thickness in the T-shaped head with arrays of 50 μm protrusions on the top. Both Alexa Fluor^™^ 488 and Alexa Fluor^™^ 350 were used for brain staining, and we successfully detected the fluorescence signal from both dyes, as shown in Fig 5a–iii. Alexa Fluor^™^ 350 exhibits relatively distinct excitation and emission wavelength peaks, with a separation of approximately 90 nm, while Alexa Fluor^™^ 488 has a narrower difference of 30 nm. In Fig 5b, the intensity difference between the control (non-stained) and experimental (stained) slices using the chips was only 6 % (T-Curl T) and 2 % (Curl T-P), respectively. The increased transmission distance for the fluorescence and the weaker fluorescence signal necessitate some adjustments. In Fig 5c–i, the channel length is aligned with the dorsal-to-ventral distance, approximately 8 mm [30]. Curl T-P demonstrates a higher intensity difference of 13 %, while T-Curl T exhibits a similar performance to the original length. The chips before and after modification are displayed in Fig 5c–ii. Subsequently, we adjusted the pore size in the porous film between the fiber-coupling hole and the micro-structured channel, increasing it from 100 μm to 300 μm. As seen in Fig 5d–i, this adjustment resulted in an approximately 7 % improvement in performance.

The enlarged pore size is noticeable in Fig 5d–ii. In general, the Curl T-P chip exhibited enhancements after optimization. The optimized chip was employed to measure stained slices under various dye concentrations to illustrate the feasibility of this preliminary study, as shown in Fig 5e. The detected intensities increased with the concentrations.

## 4. Discussion

Our work culminated in developing an ex vivo optofluidic probe following a comprehensive process of practical printing evaluation, ray tracing analysis, transmission, and fluorescence measurements. We drew from our prior T-shape designs and employed high resolution 3D printing to generate several microstructures for our printing evaluation. Enhancements in the dimensions of the sample can be achieved by refining the printing orientation and exposure parameters for optimization. Ultimately, we successfully achieved the printing of a curl T chip with a feature size of 25 μm and even incorporated secondary structures in the form of a 50 μm protrusion array on each T head.

Preceding our experimental work, ray tracing simulations were conducted to establish design guidelines and anticipate prioritizing proposed morphologies and dimensions. The results of simulations highlighted the factors, including the radial dimension (T head thickness), circumferential dimension (T width), longitudinal dimension (T length and spacing between them), and cross-section shape, significantly affecting light guidance. This influence stems from considerations such as material absorption and light refraction and reflection at the interfaces between solid structures, liquid cores, and air spaces. In practical terms, it was determined that an efficiently thin radial thickness and adequate circumferential and longitudinal dimensions could reduce the solid contact area while effectively confining the liquid core, akin to a cylinder. This configuration enhances the efficiency of light ray collection along the center axis and minimizes absorption, as validated during the evaluation of the curl T series in terms of transmission and fluorescence measurements. Among the initial seven chips, T-7 outperformed the others, particularly in transmission and fluorescence measurements, due to its moderate structural length. Subsequently, we introduced T-9 and T-10 to investigate the impact of cross-section shapes in the longitudinal direction, resulting in improved chip transmission.

Furthermore, our fluorescence measurements involving the training phantom and human plasma, mimicking a volume akin to a mouse brain, yielded results consistent with our earlier findings. This suggests that our prototype holds potential for use as an implantable probe in mouse brains as a photometry [13,31]. In addition, the results from these experiments exhibited a consistency in the performance order of the chips. For example, T-7 is identified as the best in the first group (T-1 to T-7), no matter the transmission and fluorescence measurements conducted on the lab-printed tank and the training phantom. Consequently, T-8, T-9, and T-10 were represent advancements derived from the evaluation of the initial seven chips.

We also demonstrated a non-implanted protocol by using the chip to measure the fluorescence of a mouse brain slice. However, it’s important to note that the fluorescence intensity in this scenario is weaker, given the constraints on the number of fluorescent molecules within the sliced area, their dispersion in the surrounding solutions, and the inherent brightness limitations compared to Qdots. Given these considerations, we refined our device, aligning it with the dorsal-to-ventral distance in mouse brains, guided by the design principles we summarized and the optofluidic chip we developed. This modification resulted in an approximate 20 % difference in intensity for the stained slice under the staining treatment of 100 μg ml-1 dye concentration.

In the broader context, the capabilities of these micro-structured chips were validated across various measuring environments and distinct protocols. It is important to acknowledge that the measured data can be influenced by factors such as fluorescent quenching, manual positioning errors, the distribution of fluorescent molecules, and the fluorescence background from the printing material itself, which may potentially impact the overall system performance. The next objective of the proposed device is to enhance the optical performance, such as sensitivity and transmission efficiency. The inclusion of secondary structures significantly enhances the fluorescence collection capability of our liquid core photometry. Achieving this level of performance would be extremely challenging without the use of high-resolution printing. Additionally, the biocompatibility of the current resin has yet to be fully studied, suggesting a potential need for coating the device with more biocompatible materials in the future. With the exception of optical performance, our conclusion pertains to a compact design featuring seamlessly integrated optical and microfluidic components, achieved through a one-step manufacturing process. Different from conventional solid core photometry, our device of using liquid core can facilitate drug delivery and optical sensing at the same time. Consequently, high resolution 3D printing emerges as a crucial enabler in advancing the design of miniaturized optofluidic devices. The prototype can be extended to applications in drug delivery and photomedicine treatment [32].

## Supplementary Material

Supplement

Supplementary material associated with this article can be found, in the online version, at doi:10.1016/j.snr.2024.100227.

## Figures and Tables

**Table 1:** Dimensions of T-shape structures used in simulations.

Map No.	Width [μm]	Thickness [μm]	Length [μm]	Space [μm]
a	N.A.	N.A.	N.A.	N.A.
b	654	90	350	350
c	131	90	350	350
d	393	90	350	350
e	393	190	350	350
f	524	90	350	350
g	393	90	150	350
h	430	90	350	350
i	500	90	500	350

**Table 2:** Dimensions of curl T-shape structures.

Design No.	Width [μm]	Thickness [μm]	Length [μm]	Space [μm]
T-1	N.A.	N.A.	N.A.	N.A.
T-2	393	90	350	350
T-3	654	90	350	350
T-4	524	90	150	350
T-5	524	90	750	350
T-6	524	10	350	350
T-7	524	90	350	350
T-8	524	90	350	450
T-9	524	90	350	260
T-10	524	25	350	260

## Data Availability

Data will be made available on request.
