# Supplementary material for: Ex vivo liquid core fiber photometry with high-resolution 3D printing
Source: Sens Actuators Rep. Author manuscript; Available in PMC 2025 Aug 22. (PMC12369891; doi:10.1016/j.snr.2024.100227)
Supplement: Supplement [file NIHMS2092942-supplement-Supplement.docx]

Supplementary Information

Ex Vivo Liquid Core Fiber Photometry with High-Resolution 3D Printing

Yu Chang ^a,b^, Can Wang ^a^, Ke Du ^a,^*

a. Department of Chemical and Environmental Engineering, University of California, Riverside, California 92521, United States

b. Department of Mechanical Engineering, Rochester Institute of Technology, Rochester, NY, USA 14623
E-mail: kdu@ucr.edu

In Figure S1, we build a transmission and fluorescence measurements setup featuring a lab-printed tank with replaceable solutions, a chip-fixing hole, and a sensing window.


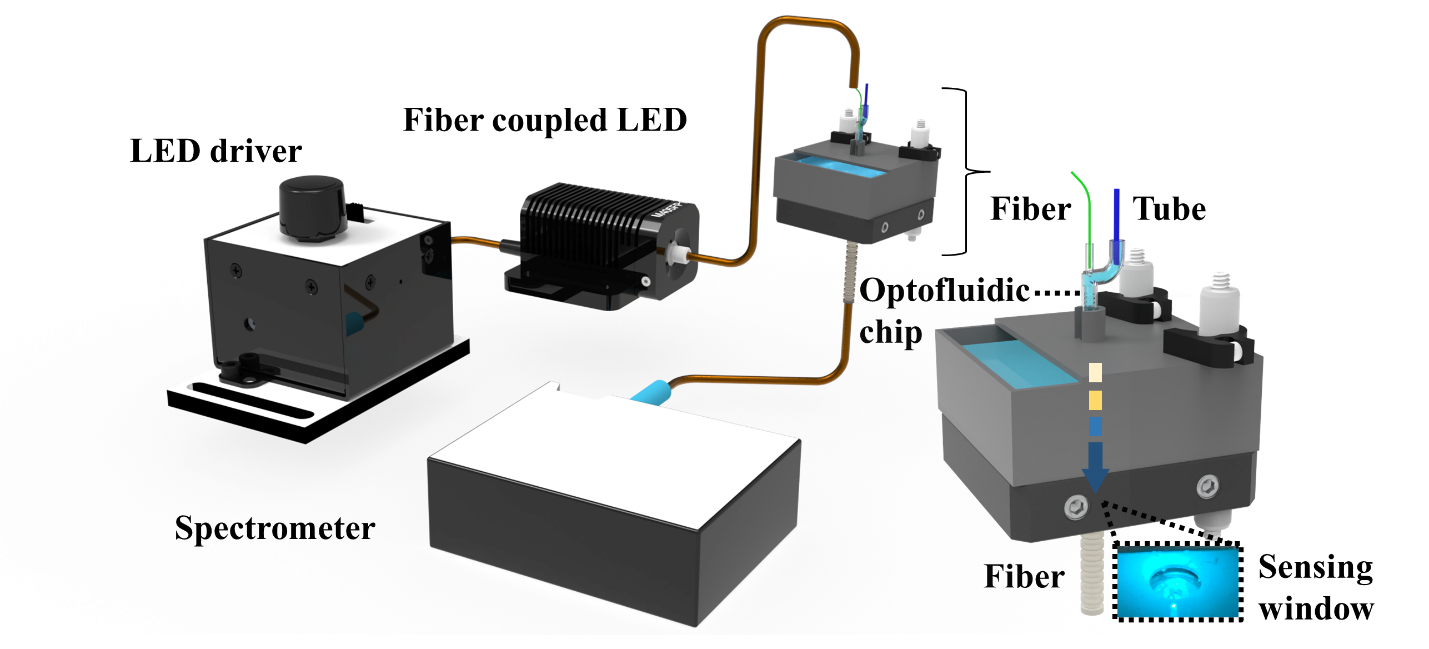


**Figure S1.** The transmission measurement setup was depicted, where the detailed information about the lab-printed tank assembly for the chip was on the lower right.

In Figure S2, the length of the optofluidic chip remained constant while the structures' dimensions and inter-spacing were varied. The chips with various dimensions were subjected to simulations and experiments to compare their performance.


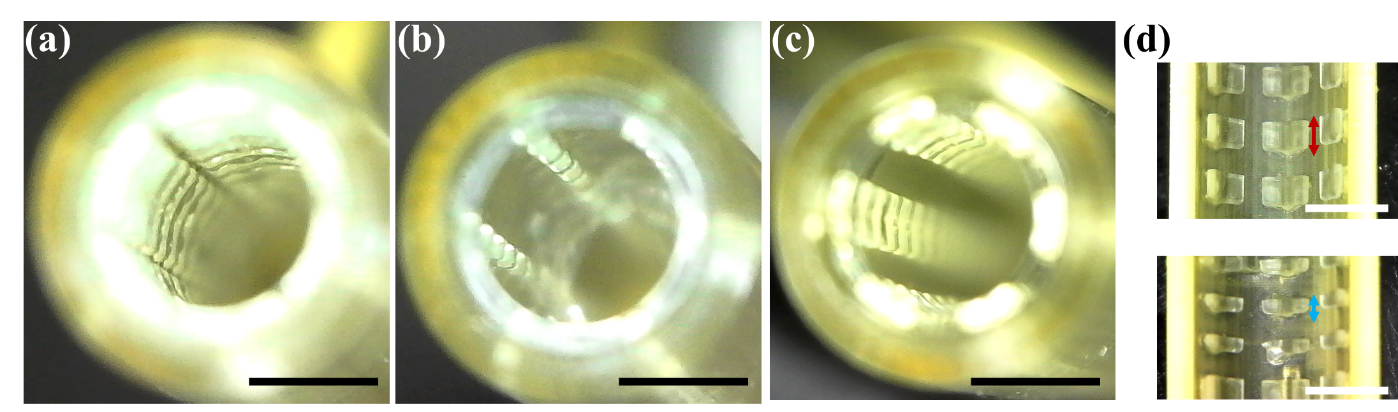
**Figure S2.** The photos represent the printed T-structures based on the dimensions used in the simulation as (a) wide T (b) narrow T (c) T with a width of 393 µm (d) various T-shape lengths. The scale bars denote 1 mm.

During the experiments, we printed the chips with different T head widths to verify if the microstructures could well support the liquid, as shown in Figure S3.


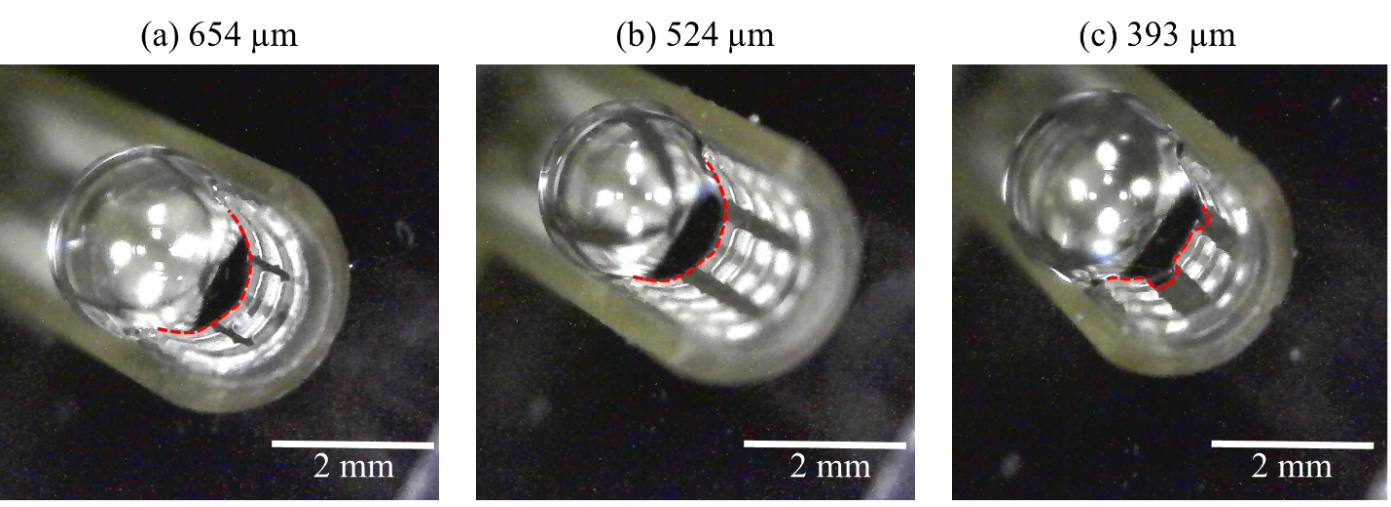


**Figure S3.** The evaluation of the water prevention scenarios among the T heads with (a) 654 µm, (b) 524 µm, and (c) 393 µm.

In Figure S4, the photo shows the T head thickness of 10 µm failed to be printed completely, thus resulting in non-superior performance in transmission and fluorescence measurements.


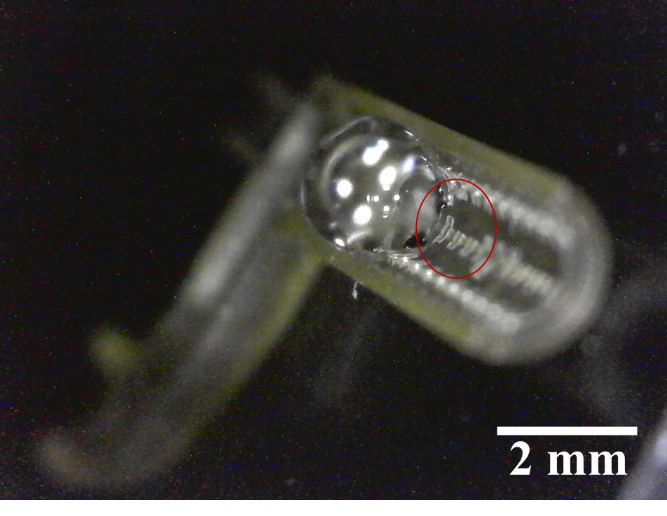


**Figure S4.** The photo of incomplete printing of the T-4 chip design (10 µm thick head).
